# VISPR-online: a web-based interactive tool to visualize CRISPR screening experiments

**DOI:** 10.1186/s12859-021-04275-5

**Published:** 2021-06-24

**Authors:** Yingbo Cui, Zihang Wang, Johannes Köster, Xiangke Liao, Shaoliang Peng, Tao Tang, Chun Huang, Canqun Yang

**Affiliations:** 1 School of Computer, National University of Defense Technology, Changsha, 410073 China; 2 College of Information Science and Engineering, Hunan University, Changsha, 410006 China; 3 Algorithms for Reproducible Bioinformatics, Institute of Human Genetics, University of Duisburg-Essen, 45147 Essen, Germany; 4 National Supercomputing Center in Changsha, Changsha, 410082 China

**Keywords:** CRISPR, CRISPR screening, Visualization, VISPR

## Abstract

**Background:**

VISPR is an interactive visualization and analysis framework for CRISPR screening experiments. However, it only supports the output of MAGeCK, and requires installation and manual configuration. Furthermore, VISPR is designed to run on a single computer, and data sharing between collaborators is challenging.

**Results:**

To make the tool easily accessible to the community, we present VISPR-online, a web-based general application allowing users to visualize, explore, and share CRISPR screening data online with a few simple steps. VISPR-online provides an exploration of screening results and visualization of read count changes. Apart from MAGeCK, VISPR-online supports two more popular CRISPR screening analysis tools: BAGEL and JACKS. It provides an interactive environment for exploring gene essentiality, viewing guide RNA (gRNA) locations, and allowing users to resume and share screening results.

**Conclusions:**

VISPR-online allows users to visualize, explore and share CRISPR screening data online. It is freely available at http://vispr-online.weililab.org, while the source code is available at https://github.com/lemoncyb/VISPR-online.

**Supplementary Information:**

The online version contains supplementary material available at 10.1186/s12859-021-04275-5.

## Background

CRISPR/Cas9, as a novel and powerful genome editing technology, mediated high-throughput screening enables systematic exploration of the functions of coding genes and non-coding elements in various contexts, including cancer, infectious disease, and development [1–6]. We previously developed MAGeCK [7] and MAGeCK-VISPR [8] to perform analysis on CRISPR screening data. To better help users explore CRISPR screening results, we developed VISPR (VISualization of crisPR screens), an interactive visualization program as part of MAGeCK-VISPR. Although VISPR enables users to examine quality control (QC) metrics and pick genes of interest, it only supports output of MAGeCK. Users should install VISPR on a local computer, and manually modify a configuration file to run the program. Data sharing between different users is also challenging in VISPR, since VISPR is designated to run on a single computer. These restrictions limit its applications.

In this work, VISPR-online, as an enhanced web-based general application for the interactive visualization of CRISPR screens, is presented to overcome the VISPR limitations. VISPR-online only requires a web browser from the client, and implements many useful functions, including an interactive display of top-ranked genes and the normalized read counts of sgRNAs. It not only supports MAGeCK, but also two more CRISPR screening analysis tools, including BAGEL [9] and JACKS [10]. Moreover, VISPR-online provides new features to display gRNA locations in a gene and save session data in the server for later retrieval. Users can either adopt the public server (http://vispr-online.weililab.org) or set up their own server from the VISPR-online source code (https://github.com/lemoncyb/VISPR-online or Additional file 1).

## Implementation

VISPR-online is implemented in “Browser-Server” mode. The frontend is implemented with HTML, jQuery, and Twitter Bootstrap, while the backend is implemented by Flask framework with Python 3. The frontend communicates with the backend by AJAX in JSON format. The gene and gRNAs location annotation are downloaded from Ensembl [11] and stored in MongoDB.

**Fig. 1:**
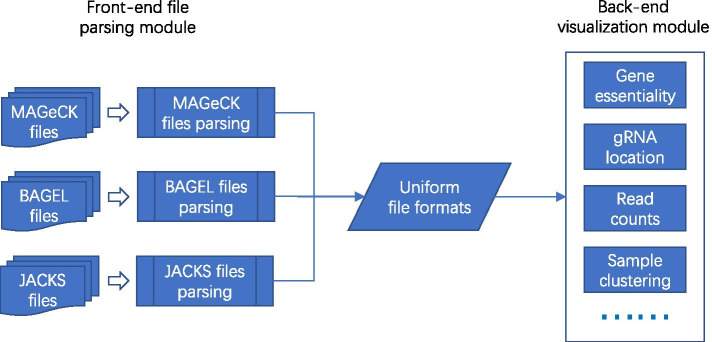
The framework of VISPR-online server side

The framework of the VISPR-online server-side is shown in Fig. 1. It mainly consists of three parts: front-end file parsing module, uniform file formats, and back-end visualization module. The file parsing module is responsible for processing files of different screening analysis tools, in which one module is implemented for each analysis tool. More screening analysis tools can be easily supported by adding more file parsing modules. The uniform intermediate file formats guarantee the excellent scalability of the front-end and back-end. The visualization module only processes the uniform file formats. Thus, it is easy to extend the functions while maintaining the stability of VISPR-online.

VISPR-online can be operated in Windows, MacOS, and Linux environments. It was tested with browsers like Safari, Chrome, and Opera. The source code and example data are provided in Additional file 1, while details of VISPR-online installation and usage are given in Additional file 2.

The input data of VISPR-online is the output of MAGeCK, MAGeCK-VISPR, BAGEL, or JACKS. VISPR-online can generate interactive statistics plots as the output to assist data analysis. Some figures can also be downloaded in SVG format.

The use cases and examples of VISPR-online are presented in the next section.

## Results and case study

**Fig. 2:**
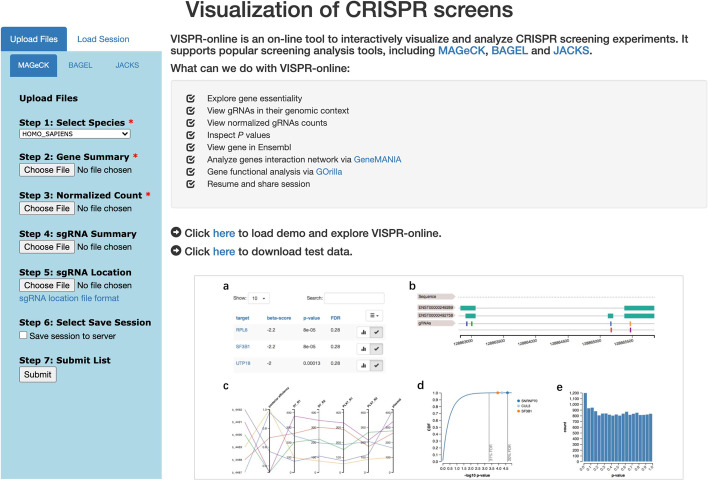
The homepage of VISPR-online. The left part shows the file uploading tab. In the right part, the top half describes the functions of VISPR-online; the middle shows the links to load demo and down example data; the lower half is scrolling pictures to show VISPR-online features

VISPR-online includes truncated sample data for demonstration purposes. The demo can be loaded directly via the button on the homepage (see Fig. 2) or the “Load Session” tab. The demo data can also be downloaded locally (see Fig. 2a) and uploaded step by step to learn about the usage of VISPR-online application.

### File upload

As shown in Fig. 2, users should select a screening analysis tool (MAGeCK, MAGeCK-VISPR, BAGEL, or JACKS) in the VISPR-online input and specify the path to the results. The corresponding relationship between the output of the analysis tool and the VISPR-online input is shown in Table 1. Users can easily find the corresponding input file by the file suffix name. The gene summary file and normalized count file are mandatory for MAGeCK and MAGeCK-VISPR. The gene summary file stores the comparison results of the screens and the rankings of genes, while the normalized count file records the normalized read count of every sgRNA in every condition. Other files are optional, including the sgRNA summary file that stores information about sgRNAs, and the sgRNA location file that records coordinates and strand of sgRNAs. Foldchange file, which contains sample foldchange information, is mandatory for BAGEL. For JACKS, gene score file and foldchange file are mandatory. The gene score of JACKS reflects the essentiality of genes.

**Table 1:** File suffix of VISPR-online input

Analysis tools	Output file	Suffix of output file	VISPR-online input
MAGeCK	Gene summary	*.gene_summary.txt	Gene summary
	Normalized count	*.count_normalized.txt	Normalized count
	sgRNA summary	*.sgrna_summary.txt	sgRNA summary
BAGEL	Foldchange	*.foldchange.txt	Foldchange
JACKS	Gene score	*_gene_JACKS_results.txt	Gene score
	Foldchange	*_logfoldchange_means.txt	foldchange

VISPR-online provides online demo loading and test data downloading. If the users prefer other data, this genome-wide screens [12] may be a good public case. The raw read counts of experiments have been provided in [12]. Users can download these count files, and analyze them with their favorite tool, such as MAGeCK, BAGEL, or JACKS. Then, they can explore the results with VISPR-online (see Table 1).

### Data visualization

**Fig. 3:**
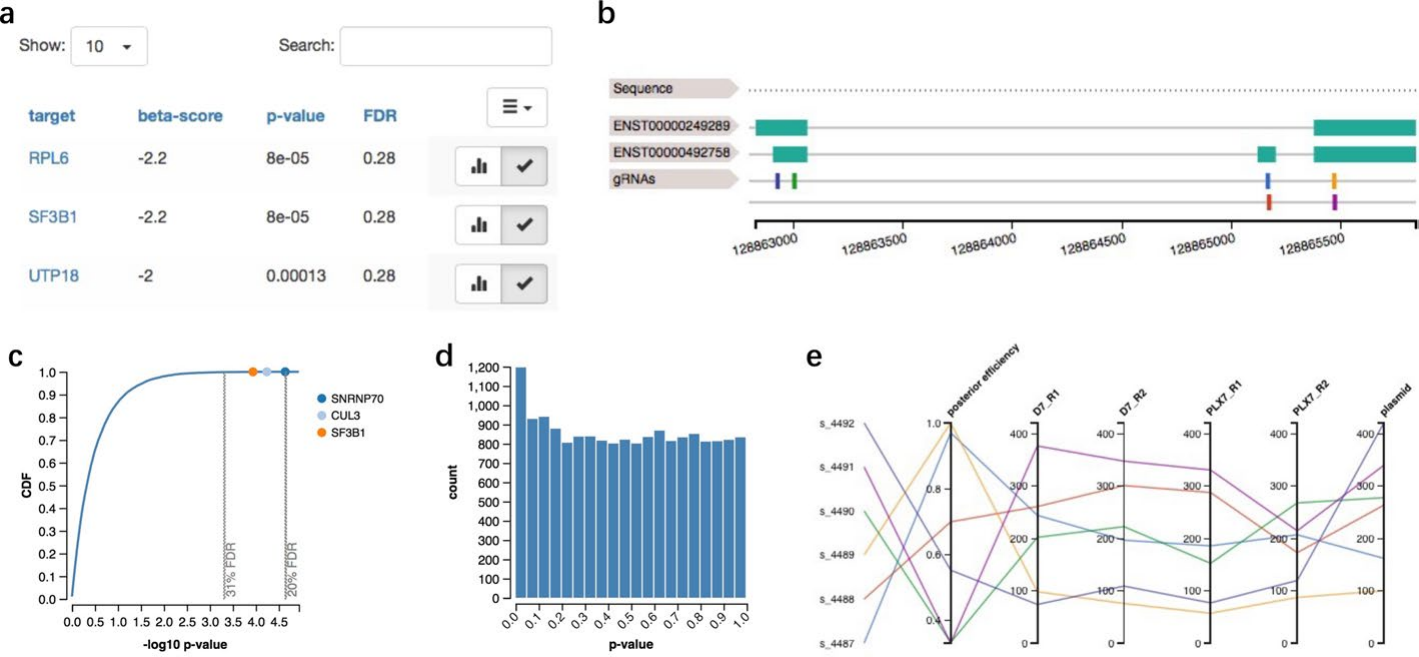
The result view of VISPR-online. The result view includes a gene exploration table (**a**), gRNAs locus of selected gene in their genomic context (**b**), normalized read counts of all samples of selected gene (**c**), distribution of *P* values as CDF plot (**d**) and histogram plot (**e**). The same gRNAs in **b** and **c** are assigned the same color

As shown in Fig. 3, once files are uploaded, VISPR-online returns the result view. The positively and negatively selected genes in each condition are presented in different tabs. In each tab, the genes’ ranking is provided in a table (Fig. 3a) in the left half of the page. The table can be sorted by any column and searched via gene names. For example, if the table is sorted by beta-score, significantly positively/negatively selected genes can be easily discovered. In MAGeCK-VISPR, beta-score measures gene selections similar to the “log fold change” in differential expression [8].

The first gene in the table is selected by default. Its sgRNAs and *p* value information are presented in the right half. If sgRNA locations are provided, the neXtprot [13] interactively zoomable feature viewer widget can be employed to display locations relative to their target genes (see Fig. 3b). All isoforms of the selected gene are listed above sgRNAs (the green bars in Fig. 3b). The sgRNA locus plot can be locally zoomed in by selection. The normalized read counts of all sgRNAs in every condition are displayed as a parallel coordinate plot (see Fig. 3c). Axes can be reordered by dragging or toggling on or off by selecting. Each sgRNA in the location plot and read count plots are assigned with the same color for ease of observation.

Besides, the distribution of *p* values is shown as a cumulative distribution function (CDF) plot in Fig. 3d and a histogram plot in Fig. 3e. Selected genes are highlighted in the CDF plot.

### Advanced features

The resulting view also provides some extra resources to explore the results. Individual genes can be viewed in Ensembl by clicking on the gene name. The GeneMANIA [14] can be employed to visualize the interaction network of selected genes, while gene function can be analyzed with Gorilla [15] as an online Gene Ontology (GO) enrichment analysis tool.

### Data retrieving and sharing

VISPR-online allows users to save data on the server and retrieve them later. As shown in Fig. 2a, if the “Save session to server” box is checked while uploading files, VISPR-online will save the whole web session to the server and return a session ID to the user. Users can resume this session later (see Fig. 2b) and share the results with collaborators using this session ID to avoid the trouble of copying data. The session ID, as a 32-bit uudi4 string, can be employed to access the data.

## Discussion

Although VISPR allows users to explore CRISPR screening results locally, but it requires manual installation and configuration. In this paper, VISPR-online is presented as an easily accessible and interactive screening visualization framework. Researchers can investigate their screening results with the web browser and easily share their findings with collaborators.

The modularity of VISPR-online makes it easy to extend functionality. We will follow the latest research of screening analysis tools, update the number of species supported every year, and integrate more valuable functions to VISPR-online to perform new tasks.

## Conclusion

VISPR-online is a general interactive framework for CRISPR screening visualization. It supports most popular screening analysis tools, including MAGeCK, BAGEL, and JACKS, while its browser interface provides various visualization features. (1) positively and negatively selected genes are displayed in separated sortable tables. (2) gRNAs are displayed in their gene context. (3) read counts of all samples are presented in parallel coordinates. (4) *p* values of selected samples are shown in CDF and histogram plots. Besides, VISPR-online provides session saving and retrieving functions. Accordingly, researchers can quickly resume their previous analysis process and share experimental discoveries with collaborators. VISPR-online is open-source, browser agnostic, and easy to install even on a laptop. More features will be added in the future versions of VISPR-online to further facilitate screening data analysis further.

### Availability and requirements

Project name: VISPR-online. Project home page: http://vispr-online.weililab.org. Operating system(s): Platform independent. Programming language: Python 3, HTML, JavaScript. Other requirements: pypi packages (flask, pymongo, PyYAML, numpy, pandas, sklearn). License: MIT https://opensource.org/licenses/MIT. Any restrictions to use by non-academics.

## Supplementary Information

**Additional file 1.** VISPR-online source code and sample data. Code and sample data used for test.

**Additional file 2.** VISPR-online installation instructions and usages.

## Data Availability

The software source code and sample data sets are included in the published article as Additional file 1 and also available on GitHub at https://github.com/lemoncyb/VISPR-online.

## Abbreviations

gRNA: Guide RNA
sgRNA: Single guide RNA
VISPR: VISualization of crisPR screens
QC: Quality control
