# Supplementary material for: VISPR-online: a web-based interactive tool to visualize CRISPR screening experiments
Source: BMC Bioinformatics. 2021 Jun 24;22:344. doi: 10.1186/s12859-021-04275-5 (PMC8223366; doi:10.1186/s12859-021-04275-5)
Supplement: Supplementary file 1 — Additional file 1. VISPR-online source code and sample data. Code and sample data used for test. [file 12859_2021_4275_MOESM1_ESM.gz › AddFile1_code-and-sample-data/master/vispr_screen/templates/qc.html]

{% extends "layout.html" %}
{% block breadcrumbs %}- {{ screen.name }}
- quality control
{% endblock %}
{% block content %}

{% if fastqc %}

#### Sequence level

{% endif %}
{% if mapstats %}

#### Read count level

{% endif %}

#### Sample level

{% endblock %}
