# Supplementary material for: VISPR-online: a web-based interactive tool to visualize CRISPR screening experiments
Source: BMC Bioinformatics. 2021 Jun 24;22:344. doi: 10.1186/s12859-021-04275-5 (PMC8223366; doi:10.1186/s12859-021-04275-5)
Supplement: Supplementary file 1 — Additional file 1. VISPR-online source code and sample data. Code and sample data used for test. [file 12859_2021_4275_MOESM1_ESM.gz › AddFile1_code-and-sample-data/master/vispr_screen/templates/compare.html]

{% extends "layout.html" %}
{% block breadcrumbs %}- compare experiments
{% endblock %}
{% block content %}

#### Target overlap

Experiments

{% for screen in screens %}
{% for condition, selections in screen.targets.items() %}
{% for selection in selections %}
{{ screen.name }} | {{ condition + " | " if condition != "default" }}{{ selection }}
{% endfor %}
{% endfor %}
{% endfor %}

Maximum FDR

{% for fdr in [0.001, 0.01, 0.05, 0.1, 0.25, 0.5] %}
{% set sel = "selected" if fdr == 0.25 else "" %}
{{ fdr }}
{% endfor %}

{% endblock %}
