# Supplementary material for: VISPR-online: a web-based interactive tool to visualize CRISPR screening experiments
Source: BMC Bioinformatics. 2021 Jun 24;22:344. doi: 10.1186/s12859-021-04275-5 (PMC8223366; doi:10.1186/s12859-021-04275-5)
Supplement: Supplementary file 1 — Additional file 1. VISPR-online source code and sample data. Code and sample data used for test. [file 12859_2021_4275_MOESM1_ESM.gz › AddFile1_code-and-sample-data/master/vispr_screen/templates/tutorial.html]

{% extends "layout.html" %}
{% block breadcrumbs %}
{% endblock %}
{% block content %}

## Quick Start Guideline

### 1. Overview of home page

VISPR-online currently supports three popular CRISPR screening analysis tools, including MAGeCK,
BAGEL and JACKS.
First, please select a tool and upload related results on the home page. If you don't have any result files,
you could load the demo or download the test data to explore VISPR-online. To upload files, please follow the steps on the left side
of the home page. If you would like to retrieve these results some time later, please check "Save
session to server" and keep the session number. Once you have a session number, you could choose "Load Session" and enter the session number
to resume the session.

### 2. Steps to upload files

Next, please upload files corresponding to each step. The asterisk indicates that the file must be uploaded. If you are not sure about the file name,
mouse over the button will prompt the suffix name of the file.
After uploading the files, you could choose whether to save the session. Finally, click the submit button to explore the results.

### 3. Resume session

If you have a session number, you could choose "Load Session" and input the session number to retreive the session. You could also click the "Load demo" button to view the sample data.

### 4. Explore the results of clustering

Options and session number are displayed at the top of the results page. We take the test data of MAGeCK as an example.
The first page is results of clustering. The clustering algorithm is k-means. We can choose different K values to
analyze the cluster heatmap.

### 5. Explore genes and gRNAs results

We could select the positive/negative selection of different samples from the drop-down menu. The results will be displayed in tables and charts. For the table, you could enter a gene name in the search box to search it. Click the column header to sort corresponding column. Click on
gene name to jump to the Ensembl page. Click the icon in the last column of the table to show gRNA locations and counts. Click the
check button to select target and highlight in p-value plot. Click the drop box in the upper right corner of the table to get more functions.
The sgRNA locus plot can be zoomed when selected with square.
For the gRNAs count chart, we could click the sample drop-down box to check the sample to be analyzed or drap to reorder them.
On the chart, we hold the axis to adjust the position. In the two graphs of distribution, we could click the middle of the graph to save the image locally.

### 6. Advanced functions

Click the icon in the upper right corner of the table, some advanced functions will show, including download table, GO enrichment, show selections
in GeneMANIA and deselect all. A GO term enrichment of the current table with GOrilla will be performed when you click GO enrichment. The page will jump to http://genemania.org/ when you click Show in GeneMANIA.

{% endblock %}
