# Supplementary material for: VISPR-online: a web-based interactive tool to visualize CRISPR screening experiments
Source: BMC Bioinformatics. 2021 Jun 24;22:344. doi: 10.1186/s12859-021-04275-5 (PMC8223366; doi:10.1186/s12859-021-04275-5)
Supplement: Supplementary file 1 — Additional file 1. VISPR-online source code and sample data. Code and sample data used for test. [file 12859_2021_4275_MOESM1_ESM.gz › AddFile1_code-and-sample-data/master/vispr_screen/templates/index.html]

{% extends "layout.html" %}
{% block breadcrumbs %}
{% endblock %}
{% block content %}

# Visualization of CRISPR screens

- Upload Files
- Load Session

- MAGeCK
- BAGEL
- JACKS

|  |
| --- |
| Upload Files |
| Step 1: Select Species \* |
| HOMO\_SAPIENS ARABIDOPSIS\_THALIANA SACCHAROMYCES\_CEREVISIAE CAENORHABDITIS\_ELEGANS DROSOPHILA\_MELANOGASTER MUS\_MUSCULUS RATTUS\_NORVEGICUS Not Sure |
| Step 2: Gene Summary \* |
|  |
| Step 3: Normalized Count \* |
|  |
| Step 4: sgRNA Summary |
|  |
| Step 5: sgRNA Location |
|  |
| sgRNA location file format |
| Step 6: Select Save Session |
| Save session to server |
| |  | | --- | | Step 7: Submit List | |

|  |
| --- |
| Upload Files |
| Step 1: Select Species \* |
| HOMO\_SAPIENS ARABIDOPSIS\_THALIANA SACCHAROMYCES\_CEREVISIAE CAENORHABDITIS\_ELEGANS DROSOPHILA\_MELANOGASTER MUS\_MUSCULUS RATTUS\_NORVEGICUS Not Sure |
| Step 2: Foldchange \* |
|  |
| Step 3: sgRNA Location |
|  |
| sgRNA location file format |
| Step 4: Select Save Session |
| Save session to server |
| |  | | --- | | Step 5: Submit List | |

|  |
| --- |
| Upload Files |
| Step 1: Select Species \* |
| HOMO\_SAPIENS ARABIDOPSIS\_THALIANA SACCHAROMYCES\_CEREVISIAE CAENORHABDITIS\_ELEGANS DROSOPHILA\_MELANOGASTER MUS\_MUSCULUS RATTUS\_NORVEGICUS Not Sure |
| Step 2: Gene Score \* |
|  |
| Step 3: Foldchange \* |
|  |
| Step 4: gRNA |
|  |
| Step 5: sgRNA Location |
|  |
| sgRNA location file format |
| Step 6: Select Save Session |
| Save session to server |
| |  | | --- | | Step 7: Submit List | |

|  |
| --- |
| Load Session |
| Step 1: Input Session No. \* |
|  |
| Load demo |
| |  | | --- | | Step 2: Load Session | |

#### VISPR-online is an on-line tool to interactively visualize and analyze CRISPR screening experiments. It supports popular screening analysis tools, including MAGeCK, BAGEL and JACKS.

#### What can we do with VISPR-online:

- Explore gene essentiality
- View gRNAs in their genomic context
- View normalized gRNAs counts
- Inspect *P* values
- View gene in Ensembl
- Analyze genes interaction network via GeneMANIA
- Gene functional analysis via GOrilla
- Resume and share session

#### Click here to load demo and explore VISPR-online.

#### Click here to download test data.

{% endblock %}
