# Supplementary material for: VISPR-online: a web-based interactive tool to visualize CRISPR screening experiments
Source: BMC Bioinformatics. 2021 Jun 24;22:344. doi: 10.1186/s12859-021-04275-5 (PMC8223366; doi:10.1186/s12859-021-04275-5)
Supplement: Supplementary file 1 — Additional file 1. VISPR-online source code and sample data. Code and sample data used for test. [file 12859_2021_4275_MOESM1_ESM.gz › AddFile1_code-and-sample-data/master/vispr_screen/templates/layout.html]

VISPR-online


Toggle navigation

VISPR-online

- Home
{% if screens and screen.name=="mle"%}- Results
{% elif screens and screen.name=="bagel"%}- Results
{% elif screens and screen.name=="jacks"%}- Results
{% endif %}- Tutorial
- FAQs

- {% block sessionnum %}
  {% endblock %}
  {% block breadcrumbs %}
  {% endblock %}

{% block content %}
{% endblock %}
