# Supplementary material for: VISPR-online: a web-based interactive tool to visualize CRISPR screening experiments
Source: BMC Bioinformatics. 2021 Jun 24;22:344. doi: 10.1186/s12859-021-04275-5 (PMC8223366; doi:10.1186/s12859-021-04275-5)
Supplement: Supplementary file 1 — Additional file 1. VISPR-online source code and sample data. Code and sample data used for test. [file 12859_2021_4275_MOESM1_ESM.gz › AddFile1_code-and-sample-data/master/vispr_screen/templates/target_clustering.html]

{% extends "layout.html" %}
{% block sessionnum %}
{% if screen.save %}- Session No: {{ screen.session }}
{% endif %}
{% endblock %}
{% block breadcrumbs %}- clustering
{% endblock %}
{% block content %}

- Clustering
{% for condition in screen.targets %}- {{ condition }}
  - Positive Selection
  - Negative Selection
{% endfor %}

Clusters

{% for k in range(2, 11) %}
{% set sel = "selected" if k == 4 else "" %}
k = {{ k }}
{% endfor %}

{% endblock %}
