# Supplementary material for: VISPR-online: a web-based interactive tool to visualize CRISPR screening experiments
Source: BMC Bioinformatics. 2021 Jun 24;22:344. doi: 10.1186/s12859-021-04275-5 (PMC8223366; doi:10.1186/s12859-021-04275-5)
Supplement: Supplementary file 1 — Additional file 1. VISPR-online source code and sample data. Code and sample data used for test. [file 12859_2021_4275_MOESM1_ESM.gz › AddFile1_code-and-sample-data/master/vispr_screen/templates/main.html]

VISPR-Online


### VISPR-Online

|  |
| --- |
| Step 1: Select Species \* |
| HOMO\_SAPIENS ARABIDOPSIS\_THALIANA SACCHAROMYCES\_CEREVISIAE CAENORHABDITIS\_ELEGANS DROSOPHILA\_MELANOGASTER MUS\_MUSCULUS RATTUS\_NORVEGICUS Not Sure |
| Step 2: Gene Summary \* |
|  |
| Step 3: Normalized Count \* |
|  |
| Step 4: sgRNAs Library |
|  |
| |  | | --- | | Step 5: Submit List | |
