# Supplementary material for: VISPR-online: a web-based interactive tool to visualize CRISPR screening experiments
Source: BMC Bioinformatics. 2021 Jun 24;22:344. doi: 10.1186/s12859-021-04275-5 (PMC8223366; doi:10.1186/s12859-021-04275-5)
Supplement: Supplementary file 1 — Additional file 1. VISPR-online source code and sample data. Code and sample data used for test. [file 12859_2021_4275_MOESM1_ESM.gz › AddFile1_code-and-sample-data/master/vispr_screen/templates/targets.html]

{% extends "layout.html" %}
{% block sessionnum %}
{% if screen.save %}- Session No: {{ screen.session }}
{% endif %}
{% endblock %}
{% block breadcrumbs %}- results
{% if condition != "default" %}- {{ condition }}
{% endif %}- {{ selection }}
{% endblock %}
{% block content %}

{% if screen.name=="mle"%}

{% if condition != "default" %}- Clustering
{% endif %}
{% for condition in screen.targets %}- {{ condition }}
  - Positive Selection
  - Negative Selection
{% endfor %}
{% endif %}
{% if screen.name=="jacks"%}

{% if selection == "genescore" %}- Gene Score
{% else %}- Gene Score
{% endif %}
{% if selection == "foldchange" %}- Foldchange
{% else %}- Foldchange
{% endif %}
{% if screen.grna!="" %}
{% if selection == "grna" %}- gRNA
{% else %}- gRNA
{% endif %}
{% endif %}
{% endif %}

×

#### Open IGV

VISPR allows you to display read counts and positions of sgRNAs in IGV.
You can open IGV directly via JAVA Web Start (not recommended on Mac OS)
or download an installable instance.

Once an IGV instance is open, you can select to load the current results with the "Load data" button below.
Finally, you can jump to each target via the
 buttons.

Open via JAVA Web Start
Download
Load data
Close

×

#### GO term enrichment with GOrilla

Perform a GO term enrichment of the currently displayed table
with the GOrilla web service.

maximum enrichment p-value

0.001
0.0001
1e-05
1e-06
1e-07
1e-08
1e-09
1e-10
1e-11

OK
Close

{% if screen.name=="mle"%}

{% else %}

{% endif %}

#### Target results

{% if screen.name=="mle"%} target | {{ "beta-score" if screen.is\_mle else "score" }} | p-value | FDR |{% else %}
{% for col in screen.targets[condition][selection][:].columns%}
{% if col !="log10-p-value" and col !="idx" %}
{% if col !="target"%} {{col}} |{% endif %}
{% endif %}
{% endfor %}
{% endif %}
{% if screen.name=="mle"%} {% if screen.is\_genes and control\_targets %}- Control genes - hide - show - show only {% endif %}- Table - Download {% if gorilla %}- GO enrichment {% endif %}- Selection - Show in GeneMANIA - Deselect all |{% endif %}

{% if screen.name=="mle"%}

{% if has\_rna\_info %}

#### gRNAs Locus

{% endif %}

#### gRNAs Count

Samples

{% for sample in samples %}
{{ sample }}
{% endfor %}
Deselect all

#### Distributions

{% else %}

#### Distributions

{% for col in screen.targets[condition][selection][:].columns%}
{% if 'Eto' in col or 'ETO' in col or 'dmso' in col or'DMSO' in col or'Dmso' in col or 'X1' in col or 'X2' in col or 'X3' in col or 'X4' in col or 'X5' in col%}{% endif %}
{% endfor %}

{% endif %}

{% endblock %}
