# Supplementary material for: VISPR-online: a web-based interactive tool to visualize CRISPR screening experiments
Source: BMC Bioinformatics. 2021 Jun 24;22:344. doi: 10.1186/s12859-021-04275-5 (PMC8223366; doi:10.1186/s12859-021-04275-5)
Supplement: Supplementary file 1 — Additional file 1. VISPR-online source code and sample data. Code and sample data used for test. [file 12859_2021_4275_MOESM1_ESM.gz › AddFile1_code-and-sample-data/master/vispr_screen/templates/faq.html]

{% extends "layout.html" %}
{% block breadcrumbs %}
{% endblock %}
{% block content %}

## FAQs:

### 1. What is VISPR-online?

VISPR-online is a web-based interactive framework for CRISPR screens exploration and visualization.
CRISPR screening helps systematically exploration of the functions of coding and non-coding elements in a genome.
We previously developed VISPR, bundled with MAGeCK,
an interactive framework to visualize CRISPR screening results
in a local computer. However, VISPR only supports output of MAGeCK, and requires manual installation and configuration.
To overcome these limitations, we developed VISPR-online, a web-based server for the visualization of CRISPR screening.
  
 The advantages of VISPR-online compared with VISPR:

- Interactive gene essentiality exploration.
- Support more popular CRISPR screening analysis tools, including
  MAGeCK,
  BAGEL, and
  JACKS.
- View gRNAs in their genomic context.
- Provide retrieving and sharing session functions.
- Installation and configuration free.

### 2. How to obtain the input files of VISPR-online?

VISPR-online is a downstream tool for MAGeCK,
BAGEL and
JACKS. The input of VISPR-online is output of
these tools. For the usage of these tools, please refer to related papers. Besides, we provide a test dataset for these three tools,
and the download link is on the main page of VISPR-online.

### 3. Format of sgRNA location file.

sgRNA location file is a tab-delimited text file without file header. Five columns are required: chromosome name,
gRNA start, gRNA stop, gRNA ID and strand. The gRNA IDs should be the same with those in read count file.

```
chr2	11143961	11143981	s_7648	-
chr3	186801168	186801188	s_46884	+
chr20	46053591	46053611	s_50992	+
chr3	46705812	46705832	s_57876	+
chr19	46634669	46634689	s_23309	-
chr7	108514201	108514221	s_42887	+
```

### 4. How to resume session and share data?

VISPR-online allows users to save sessions to the server and resume them later with session IDs.
If the "Save session to server" box is checked (Step 6 in "Upload Files" tab), the session will be saved to the server.
VISPR-online will return a session ID to the user. Users can resume the session later with the ID ("Load Session" tab).
They can also send the session ID to others to share the data.

### 5. Is VISPR-online open source?

VISPR-online is open source under MIT license.
The source code can be downloaded from GitHub.
Users can install and use VIPSR-online in a local computer or network.
The README of the repository describes how to install VISPR-online locally.

### 6. Problem caused by browser cache.

Brower usually caches the JavaScript, CSS and other data to improve speed and user experience. When
CRISP-view is updated, user needs to empty the previous cache in the browser. For Safari, press
`Opt`+`Cmd`+`E` to empty cache.

© Wei Li Lab 2019.

{% endblock %}
