# Supplementary material for: VISPR-online: a web-based interactive tool to visualize CRISPR screening experiments
Source: BMC Bioinformatics. 2021 Jun 24;22:344. doi: 10.1186/s12859-021-04275-5 (PMC8223366; doi:10.1186/s12859-021-04275-5)
Supplement: Supplementary file 2 — Additional file 2. VISPR-online installation instructions and usages. [file 12859_2021_4275_MOESM2_ESM.pdf]

VISPR-online

VISPR-online is a web-based interactive framework for CRISPR screens visualization, exploration and sharing.

Table of Contents

1. Description
2. Installation
3. Demo Test
4. FAQs
5. License
6. Contact

I. Description

VISPR-online is a web-based interactive framework for CRISPR screens visualization, exploration and sharing.

CRISPR screening helps systematically exploration of the functions of coding and non-coding elements in a genome. We previously developed MAGeCK and MAGeCK-VISPR to perform CRISPR screening data analysis. VISPR is a visualization tool included in MAGeCK-VISPR, which can be used to explore interesting genes. However, VISPR is designed for local use and some manual configutations need be set to run the program. To help the community to access the tool easily, we improve the tool and develop an online version: VISPR-online.

The advantages of VISPR-online compared with VISPR:

- Support more screening analysis tools, including MAGeCK, BAGEL and JACKS.
- Installation and configuration free. Easy to access via a browser.
- Support interactive view of sgRNA locations in the gene context.
- Enable to resume and share data and sessions.

II. Installation

VISPR-online can also be installed in a local computer or network for internal use.

Step1:

Download VISPR-online source code.

```
$ git clone https://github.com/lemoncyb/VISPR-online.git
```

Step2:

To install VISPR-online you please use the Python 3 variant of the Miniconda Python distribution (http://conda.pydata.org/miniconda.html). When installing Miniconda, make sure that you answer yes to this question:

```
Do you wish the installer to prepend the Miniconda3 install location to PATH ...? [yes|no]
```

Also, make sure that you do not have set the PYTHONPATH environment variable, because it will interfere with the Miniconda setup.

Step3:

Because the default channels of conda does not have some versions of dependent packages, add the download channels of conda by executing:

```
$conda config --add channels https://repo.anaconda.com/pkgs/free/
```

Step4:

Enter the top directory of VISPR-online:

```
$cd VISPR-online
```

In the terminal or an Anaconda Prompt, create an isolated software environment for vispr-online by executing:

```
$conda env create -f ./environment.yml
```

Note: If the error *ResolvePackageNotFound:-pandas==0.19.1* is reported, please skip to *FAQs* setup.

Step5:

Then, activate the environment by running

```
$conda activate vispr-online
```

conda activate and conda deactivate only work on conda 4.6 and later versions. For conda versions prior to 4.6, run:

Windows:

```
$activate vispr-online
```

Linux and macOS:

```
$source activate vispr-online
```

For detailed manual of miniconda, please refer to https://conda.io/projects/conda/en/latest/user-guide/tasks/manage-environments.html

III. Demo test

Step1:

Launch VISPR-online server.

Enter the top directory of VISPR-online. Execute the following script:

Windows:

```
$python ./run.py
```

Linux and macOS:

```
$ ./run.py
```

Open your browser and access http://127.0.0.1:5000. You will see the following page if successful.

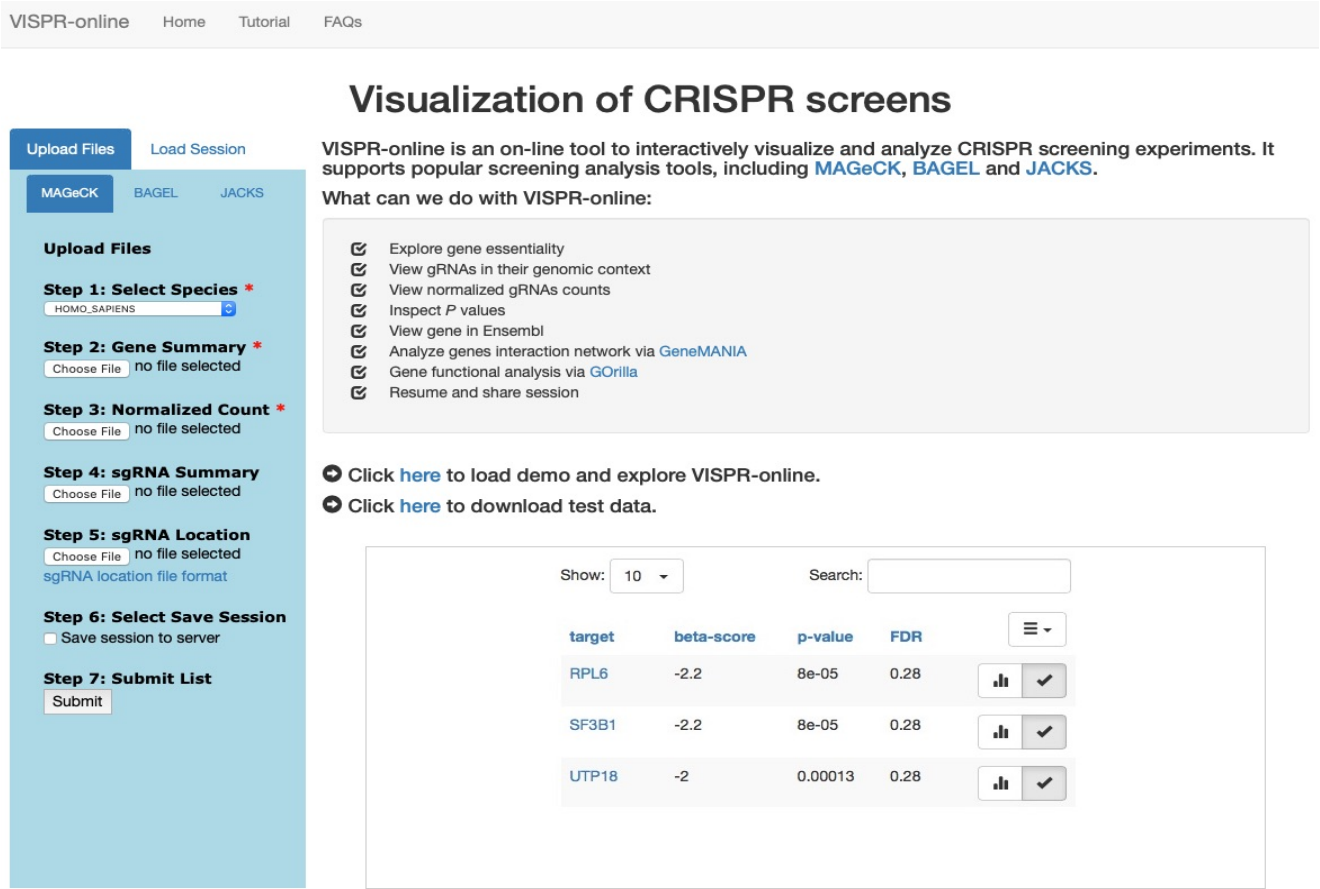

Step2:

A demo is integrated in VISPR-online repository. You could click the demo link on home page to load the project and explore the results.

Step3:

A test dataset is contained in VISPR-online repository. The path is `VISPR-online/vispr_screen/static/data/testdata.zip`. The dataset contains the following files:

- **mle.gene_summary.txt:** gene summary file.
- **all.count_normalized.txt:** normalized count file.
- **mle.sgrna_summary.txt:** sgRNA summary file.
- **sgrnas.bed:** sgRNA location file.
- **README.txt:** README file.

Note: The input of VISPR-online are direct output of screening analysis tools, so the input file types and file names are customized for different tools. Users could match files by the file suffix. For example, the name of gene summary file of MAGeCK ends with ".gene_summary.txt". Besides, the corresponding file suffix will appear when the mouse hovers over the file type, as shown in the figure below.

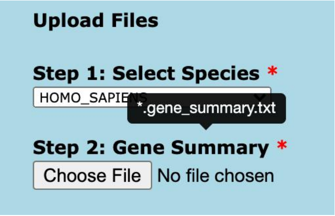

We summarize the file suffix of VISPR-online input in the table below. We also added this table and more detailed information of input file in the tutorial page.

Table 1. File suffix of VISPR-online input

Tools	Output file	Suffix of output file	VISPR-online input
MAGeCK	Gene summary	*.gene_summary.txt	Gene summary
	Normalized count	*.count_normalized.txt	Normalized count
	sgRNA summary	*.sgrna_summary.txt	sgRNA summary
BAGEL	Foldchange	*.foldchange.txt	Foldchange
JACKS	Gene score	*_gene_JACKS_results.txt	Gene score
	Foldchange	*_logfoldchange_means.txt	foldchange

Step4:

Upload corresponding files to VISPR-online. Then click the "Submit" button to explore the results. The results pages are like this.

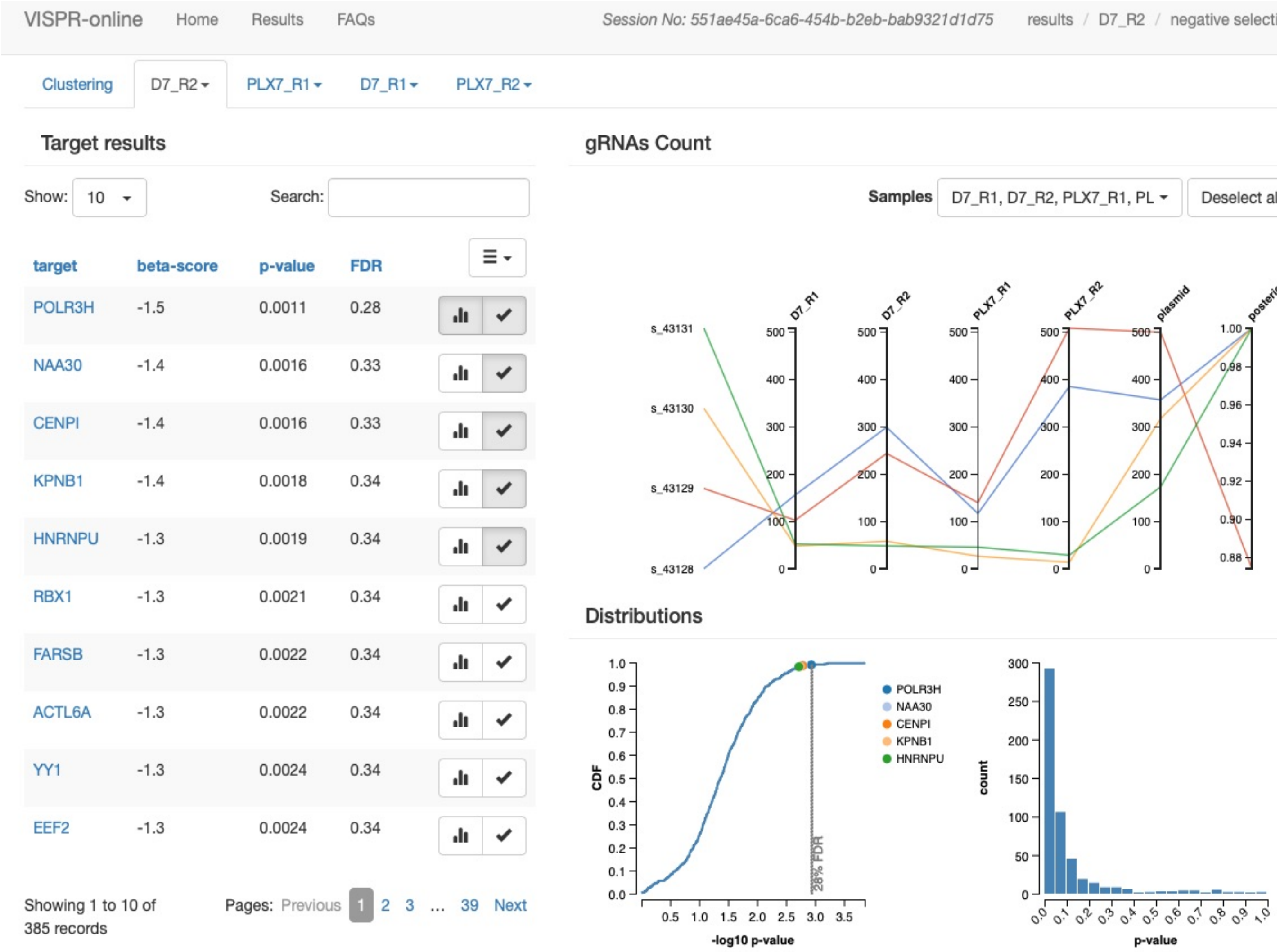

IV. FAQs

Error1:

If the error *ResolvePackageNotFound:-pandas==0.19.1* is reported, please follow the steps below to install:

Step1: Create an environment with a specific version of Python:

```
$conda create -n vispr-online python=3.5
```

Step2: Activate the environment by running

```
$conda activate vispr-online
```

Step3: Install related Python dependencies.

```
$ pip install flask
$ pip install pymongo
$ pip install PyYAML
$ pip install numpy
$ pip install pandas==0.19.1 -i http://pypi.douban.com/simple --trusted-host pypi.douban.com
$ pip install sklearn
```

Error2:

If the error *ImportError: cannot import name 'EmptyDataError' from 'pandas.io.common'* is reported, it is because the version of panda is not compatible. Please follow the above steps to install pandas *version 0.19.1*.

Error3:

The error *"/usr/bin/env: 'python': No such file or directory"* when running the run.py script. This is because the first line of the run.py script assumes that python is an alias to python3. Please install python3 to resolve this error.

License

Licensed under the [MIT license](). This project may not be copied, modified, or distributed except according to those terms.

Contact

Yingbo Cui yingbocui@nudt.edu.cn
