# Supplementary material for: VISPR-online: a web-based interactive tool to visualize CRISPR screening experiments
Source: BMC Bioinformatics. 2021 Jun 24;22:344. doi: 10.1186/s12859-021-04275-5 (PMC8223366; doi:10.1186/s12859-021-04275-5)
Supplement: Supplementary file 1 — Additional file 1. VISPR-online source code and sample data. Code and sample data used for test. [file 12859_2021_4275_MOESM1_ESM.gz › AddFile1_code-and-sample-data/master/vispr_screen/static/data/testdata.zip!/testdata/VISPR-online_manual.pdf]

VISPR-online

VISPR-online is a web-based interactive framework for CRISPR screens visualization, exploration and sharing.

Table of Contents

1. Description
2. Installation
3. Demo Test
4. License
5. Contact

I. Description

VISPR-online is a web-based interactive framework for CRISPR screens visualization, exploration and sharing.

CRISPR screen is an exciting technology to identify the functions of coding genes and non-coding genes in various contexts. We previously developed VISPR, bundled with MAGeCK, an interactive framework to visualize CRISPR screening results in a local computer. However, VISPR only supports output of MAGeCK, and requires manual installation and configuration. To overcome these limitations, here we present VISPR-online, a web-based server for the visualization of CRISPR screening.

Compared with VISPR, VISPR-online provides more useful functions:

- Interactive gene essentiality exploration.
- Support more popular CRISPR screening analysis tools, including MAGeCK, BAGEL and JACKS.
- Installation and configuration free.
- View gRNAs in their genomic context.
- Provide retrieving and sharing session functions.

Users can either use VISPR-online from the public server, or create their own servers based on VISPR-online source codes.

II. Installation

VISPR-online can also be installed in a local computer or network for internal use.

Step1:

Download VISPR-online source code.

```
$ git clone https://github.com/lemoncyb/VISPR-online.git
```

Step2:

Install Python 3. Please refer to [Python websit].

Step3:

Install related Python dependencies.

```
$ pip install flask
$ pip install pymongo
$ pip install PyYAML
$ pip install numpy
$ pip install pandas==0.19.1
$ pip install sklearn
```

III. Demo test

Step1:

Launch VISPR-online server.

Enter the top directory of VISPR-online. Execute the following script:

```
$ ./run.py
```

Open your browser and access http://127.0.0.1:5000. You will see the following page if successful.

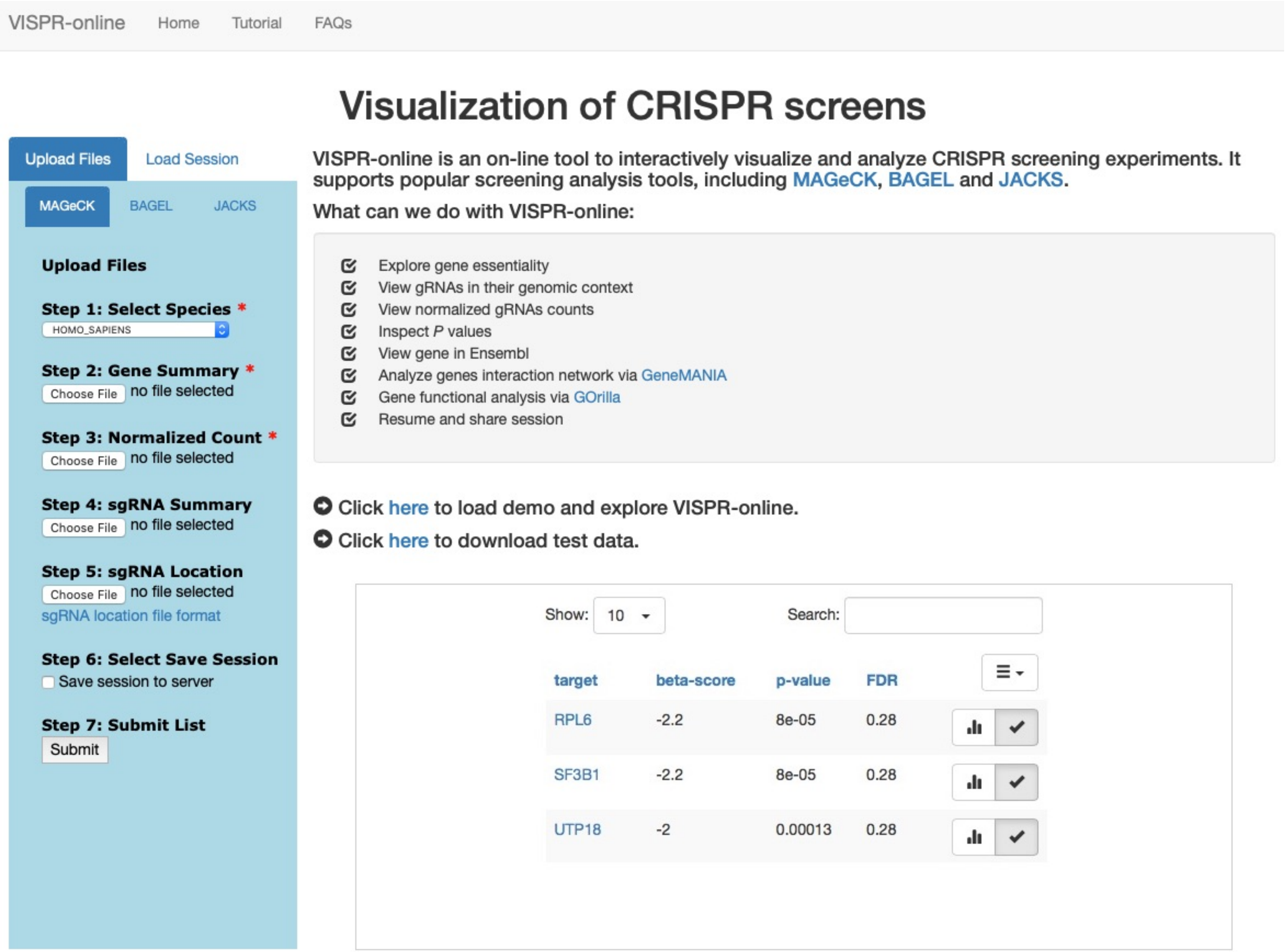

Step2:

A demo is integrated in VISPR-online repository. You could click the demo link on home page to load the project and explore the results.

Step3:

A test dataset is contained in VISPR-online repository. The path is `VISPR-online/vispr_screen/static/data/testdata.zip`.

Upload corresponding files to VISPR-online. Then click the "Submit" button to explore the results. The results pages are like this.

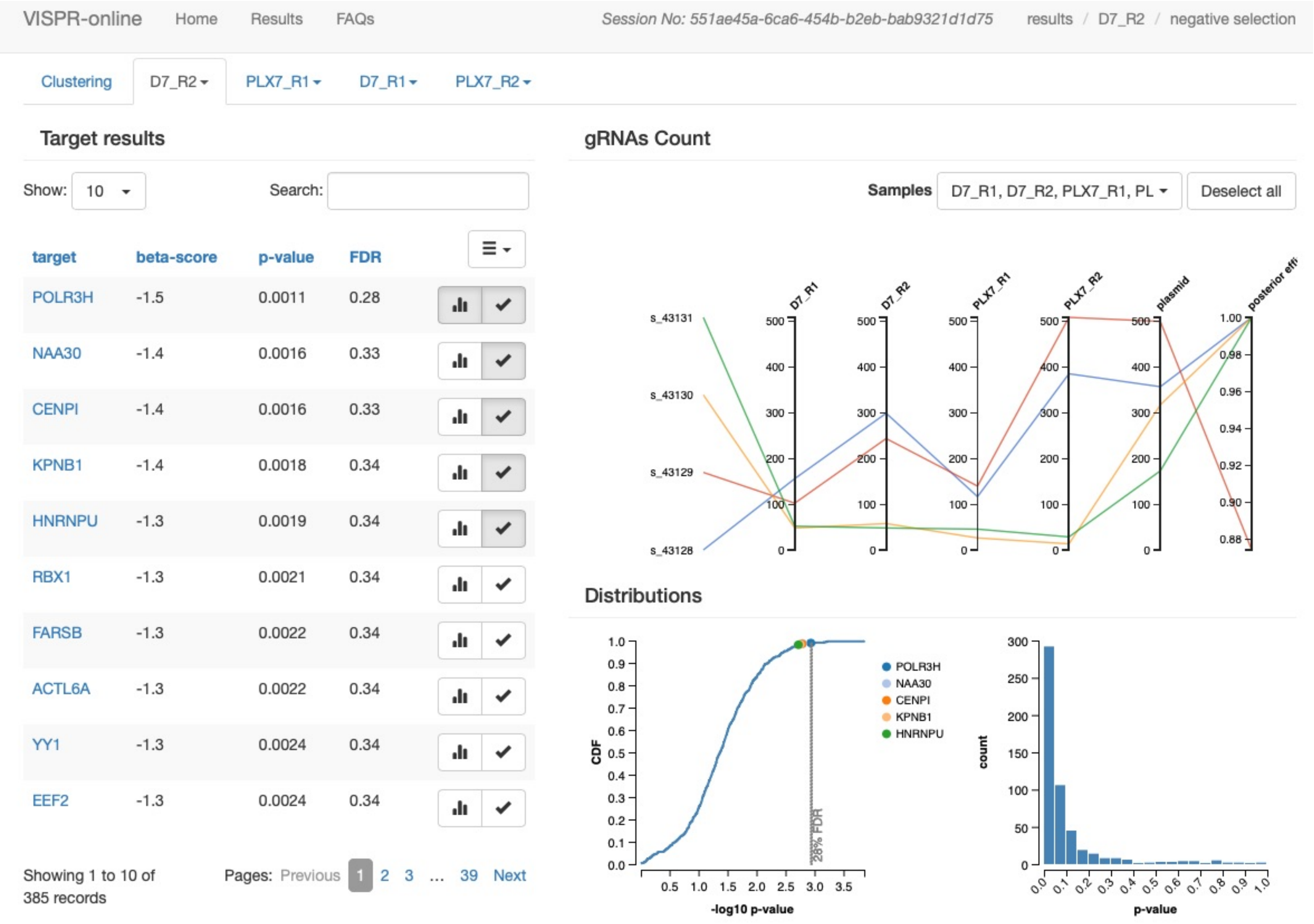

License

Licensed under the [MIT license](). This project may not be copied, modified, or distributed except according to those terms.

Contact

Yingbo Cui [yingbocui@nudt.edu.cn]()
